# Editorial: Multidimensional physiology: novel techniques and discoveries with bioimpedance measurements

**DOI:** 10.3389/fphys.2023.1243850

**Published:** 2023-06-29

**Authors:** Fernando Seoane, Lin Yang, Meng Dai, Zhanqi Zhao

**Affiliations:** ^1^ Department of Clinical Science, Intervention and Technology, Karolinska Institutet, Stockholm, Sweden; ^2^ Department of Clinical Physiology, Karolinska University Hospital, Stockholm, Sweden; ^3^ Department of Medical Technology, Karolinska University Hospital, Stockholm, Sweden; ^4^ Department of Textile Technology, University of Borås, Borås, Sweden; ^5^ Department of Aerospace Medicine, Fourth Military Medical University, Xi’an, China; ^6^ Department of Biomedical Engineering, Fourth Military Medical University, Xi’an, China; ^7^ Institute of Technical Medicine, Furtwangen University, Villingen-Schwenningen, Germany

**Keywords:** bioimpedance measurements, impedance cardiography, bioelectrical impedance analysis, electrical impedance tomography, clinical applications of bioimpedance

## Introduction

**FIGURE 1:**
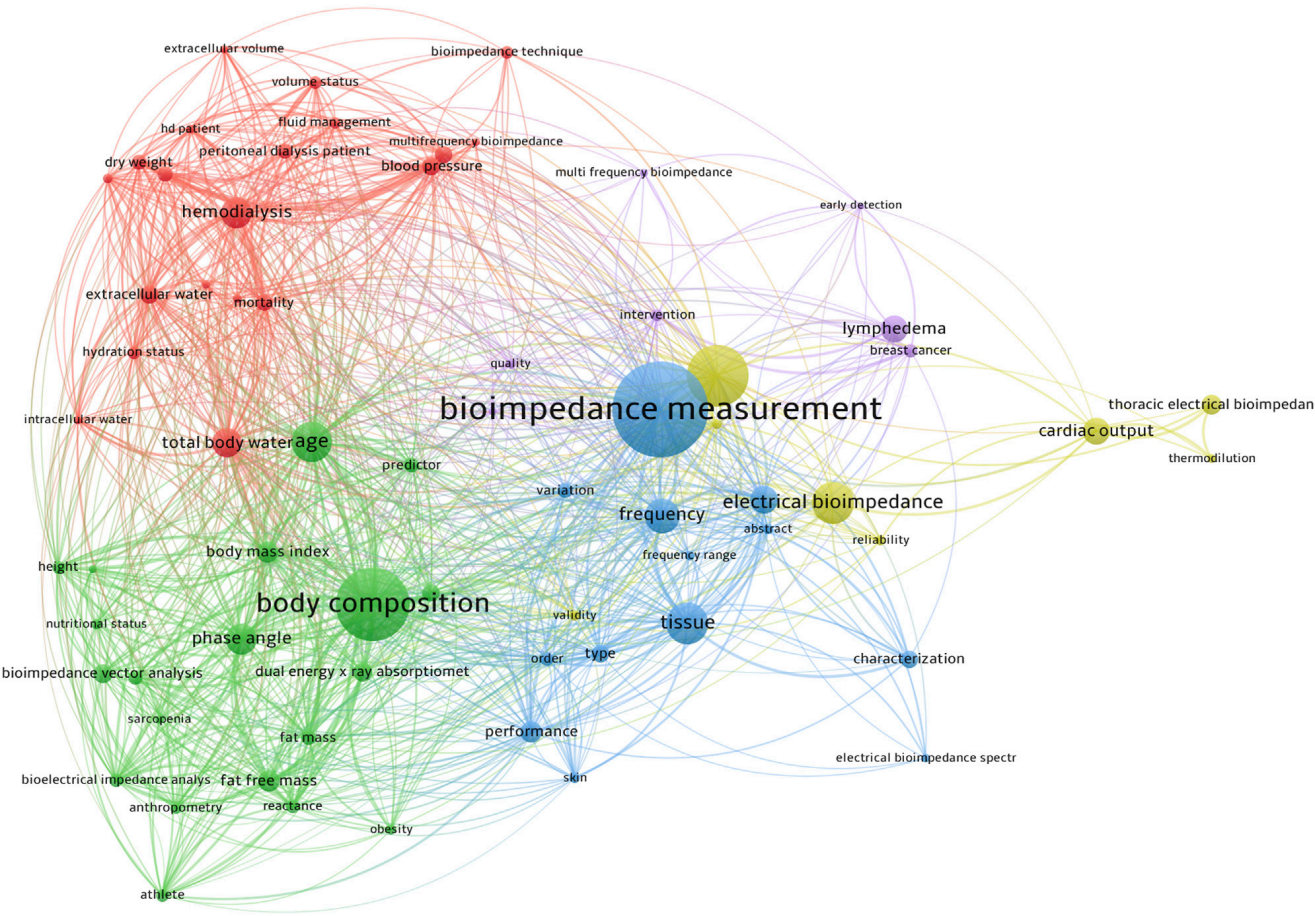
The network visualization of the keyword co-occurrence on “bioimpedance measurement” research. The color indicated various clusters; the circle size indicated the number of occurrences; the thickness of lines indicated the linkage strength; the distance between circles indicated their relationship.

Bioimpedance measurements have emerged as a powerful tool for non-invasive assessment of physiological parameters in various clinical and research applications (Khalil et al., 2014; Tortorella et al., 2023). The multidimensional nature of bioimpedance measurements allows for the evaluation of the electrical properties of tissues, providing valuable insights into different physiological processes (Figure 1). In this Research Topic entitled “*Multidimensional physiology: novel techniques and discoveries with bioimpedance measurements*,” we have seven scientific papers published. These papers cover a range of technologies, including bioelectrical impedance analysis (BIA) in general, and impedance cardiography (ICG) and electrical impedance tomography (EIT) as further developments in special directions. The findings from these papers have significant implications for both research and clinical practice.

## Research topic contributions

BIA is a well-established technique to evaluate the dielectric properties of various tissues (Kyle et al., 2004). Current research focuses on establishing the connection between the dielectric properties of tissues and clinical guidance. Wang et al. from Xi’an explored the temperature dependence in the dielectric properties of blood. An impedance measurement box with high heat transfer efficiency was constructed, and the frequency they examined ranged from 10 Hz to 1 MHz. The study provides valuable insights into the biological effects of electromagnetic fields and temperature-related variations in disease diagnosis and treatment. Understanding these temperature characteristics contributes to the development of more accurate models and measurement techniques.

In a retrospective study, Lee and Kim investigated the correlation between the phase angle obtained through BIA and the inflammatory markers after abdominal surgery in 221 patients. They examined the potential of using phase angle as a new biomarker for early detection and treatment of infections. The results suggest that phase angle could be used as a valuable predictor of postoperative infections that only in female patients. Nevertheless, by incorporating phase angle measurements into routine monitoring, healthcare professionals can identify patients at risk of developing infections sooner, enabling timely intervention and improved patient outcomes.

Another investigation (Wang et al.) from Xi’an used BIA to discriminate normal renal tissue from renal cell carcinoma. The dielectric properties of the human renal tissue of 69 cases were measured after tissue isolation. The results showed the potential to revolutionize cancer diagnosis and treatment. By leveraging the differences in impedance parameters and characteristic frequencies, clinicians may be able to detect renal cell carcinoma at an early stage, leading to timely interventions and improved patient outcomes.

ICG has been used for decades to assess the cardiac output and heart function (Siedlecka et al., 2015). However, challenge still exists in identifying all the typical characteristic points (Benouar et al., 2018). Benouar et al. presented a predictive model for forecasting ICG missing points. Using previously recorded datasets, the model was trained and validated. By predicting missing data points in the ICG complex, this model enhances the accuracy of hemodynamic parameter assessment, thereby improving the quality of patient care and decision-making.

EIT is a non-invasive imaging modality. In recent years, EIT has gained more attention from physicians. In this Research Topic, 3 papers related to this imaging technique are published. Qin et al. summarized the EIT hardware publications in the past 3 decades. The advancements in EIT hardware highlighted in this bibliometric analysis lay the foundation for improved system performance and optimization, enabling developers who are newly joining this field to have a better view of precise and reliable bioelectrical impedance imaging. This opens up new possibilities for a range of clinical applications, including lung ventilation monitoring, cardiac imaging, and tumor detection.

Wang et al. from Zhejiang and Shanghai presented a clinical observational study using pendelluft as a predictor of weaning outcome in critically ill patients. A total of 60 patients were evaluated at 4 different time points. They found that by monitoring pendelluft volume, clinicians could better assess a patient’s readiness for extubation, potentially reducing the risk of weaning failure and improving the overall success of the weaning process. This study adds to our understanding of respiratory dynamics during spontaneous breathing trials.

Ouypornkochagorn et al. focused on continuous monitoring of hemorrhagic stroke patients using EIT. In this simulation study, the authors investigated the previously proposed method in simulated clinical settings and demonstrated the potential clinical application of EIT for long-term monitoring of hemorrhagic stroke patients. By providing real-time information on cerebral hemodynamics, EIT might aid in assessing stroke severity, guiding treatment decisions, and monitoring the effectiveness of interventions, which requires further validations.

## Future and potential developments

The findings from these seven papers pave the way for exciting future developments in the field of bioimpedance measurements. Some potential areas for future exploration include the following.

Integration of multimodal approaches: Since bioimpedance measurements provide different angles of characterizing tissue properties, researchers can further investigate the integration of bioimpedance measurements with other modalities, such as imaging techniques, spectroscopy, and genetic markers. For example, computed tomography provides anatomical priors that can be used for lung function monitoring with EIT (Yang et al., 2021a). Another example is to combine the information from ultrasound and EIT for clinical decision-making (Wang et al., 2022). Combining multiple data sources could provide a comprehensive understanding of physiological processes and enable more accurate diagnosis and treatment strategies.

Advancements in machine learning and artificial intelligence: The application of machine learning and artificial intelligence algorithms to bioimpedance data holds immense potential. Several recent publications highlight the use of machine learning and artificial intelligence to analyze the measurements and provide useful feedback (Rixen et al., 2022; Baran et al., 2023; Yang et al., 2023). By leveraging these techniques, researchers can develop predictive models, identify complex patterns, and enhance decision-making in clinical settings.

Point-of-care applications: The development of portable and user-friendly bioimpedance devices could revolutionize point-of-care applications (Yang et al., 2021b; Zouari et al., 2022). These devices can enable quick and non-invasive assessments of various conditions, improving access to healthcare and facilitating early detection and intervention.

Personalized medicine: Bioimpedance measurements offer the potential for personalized medicine by providing insights into an individual’s unique physiological characteristics. Recent randomized-controlled studies suggested that it could change the clinical outcome significantly compared to traditional methods (He et al., 2021; Hsu et al., 2021). With further research, these measurements can inform tailored treatment plans, optimize drug dosages, and guide interventions for optimal patient outcomes.

Long-term monitoring: Continuous monitoring using bioimpedance techniques, as demonstrated in the paper from Ouypornkochagorn et al., can be extended to other medical conditions. Long-term monitoring can provide valuable data on disease progression, treatment response, and overall patient wellbeing.

In conclusion, the present Research Topic highlights the remarkable advancements and potential future developments in bioimpedance measurements. The improvements in hardware, understanding of temperature-dependent properties, exploration of novel biomarkers, development of predictive models, and integration with other modalities all contribute to the growing body of knowledge in this field. As interdisciplinary collaborations continue and further research unfolds, the future of bioimpedance measurements promises transformative advancements in healthcare, enhancing patient outcomes and paving the way for personalized and data-driven medicine.
